# Inhibition of Heterogeneous Nucleation in Water by Hydrogel Coating

**DOI:** 10.34133/research.0190

**Published:** 2023-07-06

**Authors:** Siyang Li, Panpan Zhu, Yaoting Xue, Lei Wang, Tuck-Whye Wong, Xuxu Yang, Haofei Zhou, Tiefeng Li, Wei Yang

**Affiliations:** ^1^ Department of Engineering Mechanics, Zhejiang University, Hangzhou 310027, China.; ^2^ Key Laboratory of Soft Machines and Smart Devices of Zhejiang Province, Zhejiang University, Hangzhou 310027, China.; ^3^ Center for X-Mechanics, Department of Engineering Mechanics, Zhejiang University, Hangzhou 310027, China.; ^4^ School of Biomedical Engineering and Health Sciences and Advanced Membrane Technology Research Centre, Universiti Teknologi Malaysia, Skudai 81310, Malaysia.

## Abstract

Heterogeneous nucleation plays a critical role in the phase transition of water, which can cause damage in various systems. Here, we report that heterogeneous nucleation can be inhibited by utilizing hydrogel coatings to isolate solid surfaces and water. Hydrogels, which contain over 90% water when fully swelled, exhibit a high degree of similarity to water. Due to this similarity, there is a great energy barrier for heterogeneous nucleation along the water–hydrogel interface. Additionally, hydrogel coatings, which possess polymer networks, exhibit higher fracture energy and more robust adhesion to solid surfaces compared to water. This high fracture and adhesion energy acts as a deterrent for fracture nucleation within the hydrogel or along the hydrogel–solid interface. With a hydrogel layer approximately 100 μm thick, the boiling temperature of water under atmospheric pressure can be raised from 100 to 108 °C. Notably, hydrogel coatings also result in remarkable reductions in cavitation pressure on multiple solid surfaces. We have demonstrated the efficacy of hydrogel coatings in preventing damages resulting from acceleration-induced cavitation. Hydrogel coatings have the potential to alter the energy landscape of heterogeneous nucleation on the water–solid interface, making them an exciting avenue for innovation in heat transfer and fluidic systems.

## Introduction

Heterogeneous nucleation, which has a preference for occurring along the water–solid interfaces [1–3], is a phenomenon that is widespread in nature. For instance, during transportation, water in trees cavitates under negative pressure and subsequently causes embolism [4–6]. The snapping shrimp creates cavitation in water around its claw and utilizes the energy pulse during the collapse of cavitation bubbles to stun and kill prey animals [7,8]. Thermal springs discharge boiled groundwater that is heated by volcanic activity, which generates geothermal energy for greenhouses [9,10]. In various engineering applications, including heat transfer [11,12], water transportation [5,13,14], and propulsion systems [15,16], the phenomenon of heterogeneously nucleated cavitation and boiling carries significant impact. This is because bubble nucleation carries substantial energy, alters the efficiency of heat and mass transfer, and causes damage to neighboring solids. For example, in the cooling system of a nuclear reactor, nucleation can adversely affect the performance of coolant circulation and heat exchangers. In transportation systems, cavitation leads to pipeline blockage and leakage. In high-velocity flow, cavitation causes surface damage and component failure in propellers and pumps. Considering the wide-ranging implications across various disciplines, it is of great significance to push the boundary of fundamental science to inhibit nucleation.

The presence of nucleation sites is critical for both cavitation and boiling processes. To hinder the occurrence of heterogeneous nucleation, one can decrease the number of such sites by modifying the surface morphology of the solid material that comes into contact with water, such as by enhancing its hydrophilicity [17] and smoothness [18]. A hydrophilic surface attracts water molecules, making it more difficult to detach them from the surface. On the other hand, a smooth surface is less likely to trap bubbles that can act as nuclei and continuously grow. Although various surface treatment methods have been investigated, water contained in a vessel can only be slightly superheated above its saturation temperature by 2 to 3 °C at atmospheric pressure [19]. Cavitation occurs at a pressure near the saturated vapor pressure [20,21], while nucleation sites can be eliminated in specific conditions, such as by heating water in a host liquid, which can raise its superheat temperature up to 279.5 °C [22], or by adding water to a mineral to achieve a water tension of −120 MPa [23,24]. Heterogeneous nucleation of water is inevitable under practical circumstances. Therefore, a stable solid surface that is completely wet and resembles a liquid is yet to be proposed as a practical approach to prevent heterogeneous nucleation.

The hydrogel is a polymeric substance that can contain more than 99% water when fully swelled [25]. It possesses favorable mechanical, electrical, and biological properties and finds application in tissue engineering [26] and flexible electronics [27,28], among other fields. Unlike other solid substances that have their hydrophilicity enhanced by nanoparticle coating [17,29,30], chemical grafting [31,32], and physical treatment [33–35], hydrogel shares extremely similar characteristics with water, rendering its surface highly hydrophilic. Furthermore, hydrogel can be produced from a precursor to attain a liquid's surface smoothness, which is superior to the smoothness achieved by traditional polishing [18], sintering [36], electrochemical deposition [37], and similar methods used for solids. Contrary to treated hydrophilic surfaces that may revert to hydrophobic ones over time and smooth surfaces that are prone to scratching [12,35], the hydrogel surface is stable and maintains its properties even when subjected to stretch, scratch, swell, and slide [38].

In accordance with the reasoning stated above, we propose inhibiting the heterogeneous nucleation of water via hydrogel coating. We coated solid substrates with adhesion interfaces using silane chemistry and analyzed the hydrophilicity, roughness, fracture energy, and adhesion energy of the hydrogel coating, which effectively elevated the energy barrier of heterogeneous nucleation. Notably, a remarkable reduction in the cavitation pressure of water was observed on various substrates coated with hydrogel. Furthermore, an increase in the emergent superheat temperature of water was detected at atmospheric pressure on hydrogel-coated glass. The durability of the hydrogel coating was demonstrated by measuring the surface roughness before and after a rub test. The hydrogel coating's ability to inhibit nucleation can be applied to various systems to prevent damage caused by explosive boiling and cavitation.

## Results

### Mechanism of inhibiting nucleation by hydrogel coating

According to the classical theory of nucleation, a certain amount of energy is necessary to initiate nuclei in water [4,39]. The energy barrier can be determined by the equation: $\text{∆}F=\frac{4}{3}\pi {R_{c}}^{2}\sigma f\left(\theta ,,,\beta ,,,R_{0}\right)$, where *R*_c_ represents the critical radius of a bubble in water that marks the point of bubble expansion. *σ* denotes the amount of energy required to create a unit area surface, which is generally equivalent to the surface energy of water. On the other hand, *f*(*θ*, *β*, *R*_0_) indicates the prefactor of the energy required for nucleation when the process is heterogeneous. *θ* is the contact angle of water on the substrate, while the geometry takes the form of a cone with a semi-angle of *β* if the substrate surface is rough. *R*_0_ refers to the radius of the small bubble that is formed at the interface. You may refer to the Supplementary Materials for further details on the derivation of the energy barriers based on classical nucleation theory.

**Fig. 1.**
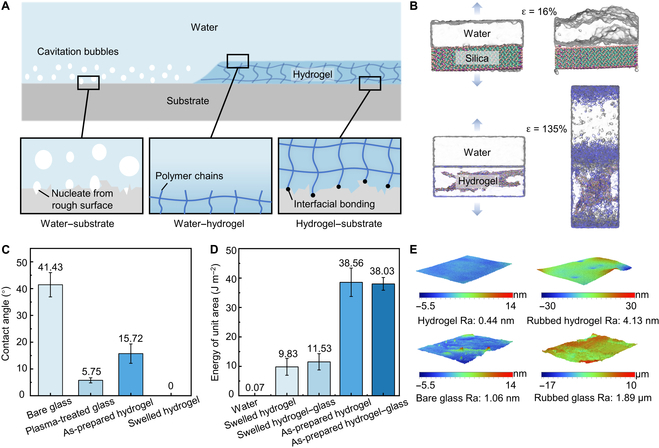
Mechanism of inhibiting nucleation in water by hydrogel coating. (A) The interfaces with and without hydrogel coating that may generate nucleation in heterogeneous systems. (B) Snapshots of AAMD simulations of water and silica or water-soluble polymer network of hydrogel at the initial and nucleation states. The upper part is a system of water and silica, while the lower part is composed of water and hydrogel. The white transparent part represents water. (C) The contact angle illustrates the hydrophilicity of bare, plasma-treated, as-prepared hydrogel-coated, and fully swelled hydrogel-coated glass. (D) The fracture energy of the as-prepared hydrogel, fully swelled hydrogel, and their adhesion energy to glass. (E) Three-dimensional surface profiles of the hydrogel coating and glass before and after rubbing (94 μm × 125 μm).

A hydrogel coating alters the energy landscape of nucleation in a water-containing system (Fig. 1A and Fig. S1). In the absence of a hydrogel coating, nucleation may take place in water or at the water–substrate interface (Fig. S1A). Since the prefactor *f*(*θ*, *β*, *R*_0_) <<1, water requires minimal energy deposition along the water–substrate interface. Conversely, with the presence of a hydrogel coating, nucleation may occur at various sites, such as in water, at the water–hydrogel interface, in the hydrogel, or at the hydrogel–substrate interface (Fig. S1A). (a) At the water–hydrogel interface, hydrogels contain water-soluble polymer chains that exhibit a soft and aqueous nature, with a remarkably smooth (*β* approaches π/2) and hydrophilic (*θ* approaches 0) surface. With a high water content of no less than 90%, the liquid–vapor energy on the hydrogel surface approaches the surface energy of water (*σ*_water–hydrogel_ ≈ *σ*_water_ =7.28 × 10^−2^ J m^−2^). Consequently, the prefactor value of *f*_water–hydrogel_ >> *f*_water–substrate_, and a higher energy is required to nucleate vapors at the water–hydrogel interface as compared to the water–substrate interface. (b) In hydrogels, when the cavity size is smaller than the mesh size (~10 μm), the energy involves both the surface energy of water and the elastic deformation of polymer chains during cavitation initiation. When the cavities reach sizes capable of inducing polymer chain rupture, the energy required for the creation of a unit area surface for homogeneous nuclei includes both the surface energy of water and the fracture energy of polymer networks (10 to 100 J m^−2^) [40]. We have *σ*_hydrogel_ >> *σ*_water_, hence the energy barrier for nucleation in hydrogels is much greater than that in water [41]. (c) At the hydrogel–substrate interface, the prefactor value of *f*(*θ*, *β*, *R*_0_) remains constant. When the hydrogel strongly adheres to the substrate, the adhesion energy obeys *σ*_adhesion_ ≈ *σ*_hydrogel_ >> *σ*_water_ [42]. Consequently, vapor nucleation at the hydrogel–substrate interface also requires more energy than that at the water–substrate interface. Nucleation may occur at both the water–hydrogel interface and the hydrogel–substrate interface. With the application of a hydrogel coating, the lower limit of the nucleation energy barrier is considerably increased, leading to nucleation suppression. Figure 1B illustrates the cross-sectional snapshots of the atomic structures for the water–silica substrate and water–hydrogel systems at their initial states and the moments when nucleation occurs. An evident nucleus is observed at the interface between water and silica substrate when the strain reaches 16% (upper part of Fig. 1B), while the cavitation generated at the water–hydrogel interface or in water requires 135% strain (lower part of Fig. 1B).

### Performance of the hydrogel coating

We applied a silane chemistry approach [43–45] to coat hydrogels onto adhesive substrates. The hydrogel precursor was produced through free radical polymerization of acrylamide (AAm) with 3-(trimethoxysilyl) propyl methacrylate, resulting in a chain-like structure. Over time, the precursor solidifies into a hydrogel through silanol group condensation and polymer chain cross-linking. Additionally, the silanol groups on the hydrogel also condense with the hydroxyl groups on the substrate, ensuring interfacial adhesion (Fig. S2). The as-prepared hydrogel coating displays a water content of 85.73%, which swells to 94.15% when fully immersed in pure water for 48 h (Fig. S3).

We characterized the hydrophilicity, roughness, and fracture energy of the hydrogel coating, which contributed to *θ*, *β*, and *σ* and consequently affected the nucleation energy barrier. We employed glasses as the model substrates. Prior to and after plasma treatment or hydrogel coating (Fig. 1C and Fig. S4), we measured the contact angle of the glass. Plasma treatment boosted the glass's hydrophilicity (contact angle decreases from 41.43° ± 4.54° to 5.75° ± 0.94°). The as-prepared hydrogel coating demonstrated a contact angle of 15.72 ± 3.61°, which was less hydrophilic than plasma-treated glass. When the hydrogel swells, its water content increases, and its contact angle decreases dramatically to 8.49° ± 1.49° at 88.69% water content, and reaches 0° at 90.68% water content (Movie S7). Further swelling of the hydrogel to the equilibrium state does not affect its hydrophilicity (Fig. S4). Through a 90° peeling test, we determined the fracture energy of the hydrogel to be 38.56 J m^−2^ for the as-prepared hydrogel and 9.83 J m^−2^ for the fully swelled hydrogel. Their adhesion energies to glass were comparable to their fracture energies, which is proven by the observation of cohesive failure. The actual adhesion energy is anticipated to be higher and large enough to adhere well to the substrates. Moreover, the hydrogel's fracture and adhesion energies surpassed the surface energy of water by orders of magnitude (Fig. 1D). The hydrogel coating was derived by naturally smoothing its precursor over the glass, and the as-prepared hydrogel succeeded in retaining the precursor's surface smoothness with an arithmetic average roughness of 0.85 nm (Ra = 0.85 nm) (Fig. S5). Hydrogel swelling further enhanced the smoothness to Ra = 0.44 nm. Compared to the hydrogel coating, a bare transparent glass (Ra = 1.06 nm) was slightly rougher (Fig. 1E). During the change in water content, the mechanical properties of the hydrogel including its modulus and toughness change, but the hydrogel surface retains its high smoothness and hydrophilicity, thus preserving its cavitation inhibition capability. Rubbing would ruin the surface of solids. We delicately rubbed sandpaper against glasses and hydrogels, increasing the Ra of a glass by 3 orders of magnitude, while the Ra of a hydrogel increased by only 1 order of magnitude and reached the same level as an unrubbed glass. Hydrogels have high water content that lubricates the surface and viscoelasticity that conforms to the shape of the contact. They can fill in micro-irregularities, increase contact area, and disperse stress. This reduces pressure and friction at the surface contact area. A continuous rub test by a rheometer clarified the hydrogel's resistance to friction. During 3 h of rubbing, the surface of the hydrogel coating was undamaged and its friction coefficient maintained between 0.04 to 0.06 (Fig. S7).

### Heterogeneous nucleation phenomenon

Prior to the process of nucleation, water attains a metastable state via 2 distinct mechanisms: stretching below its saturated vapor pressure and superheating beyond its boiling temperature [1,46]. Subsequent to the occurrence of nucleation, water undergoes a phase transition by means of cavitation or boiling, and eventually, it reverts to the state of equilibrium. Through the obstruction of nucleation using a hydrogel coating, the cavitation pressure may be reduced while simultaneously the boiling temperature may be increased.

**Fig. 2.**
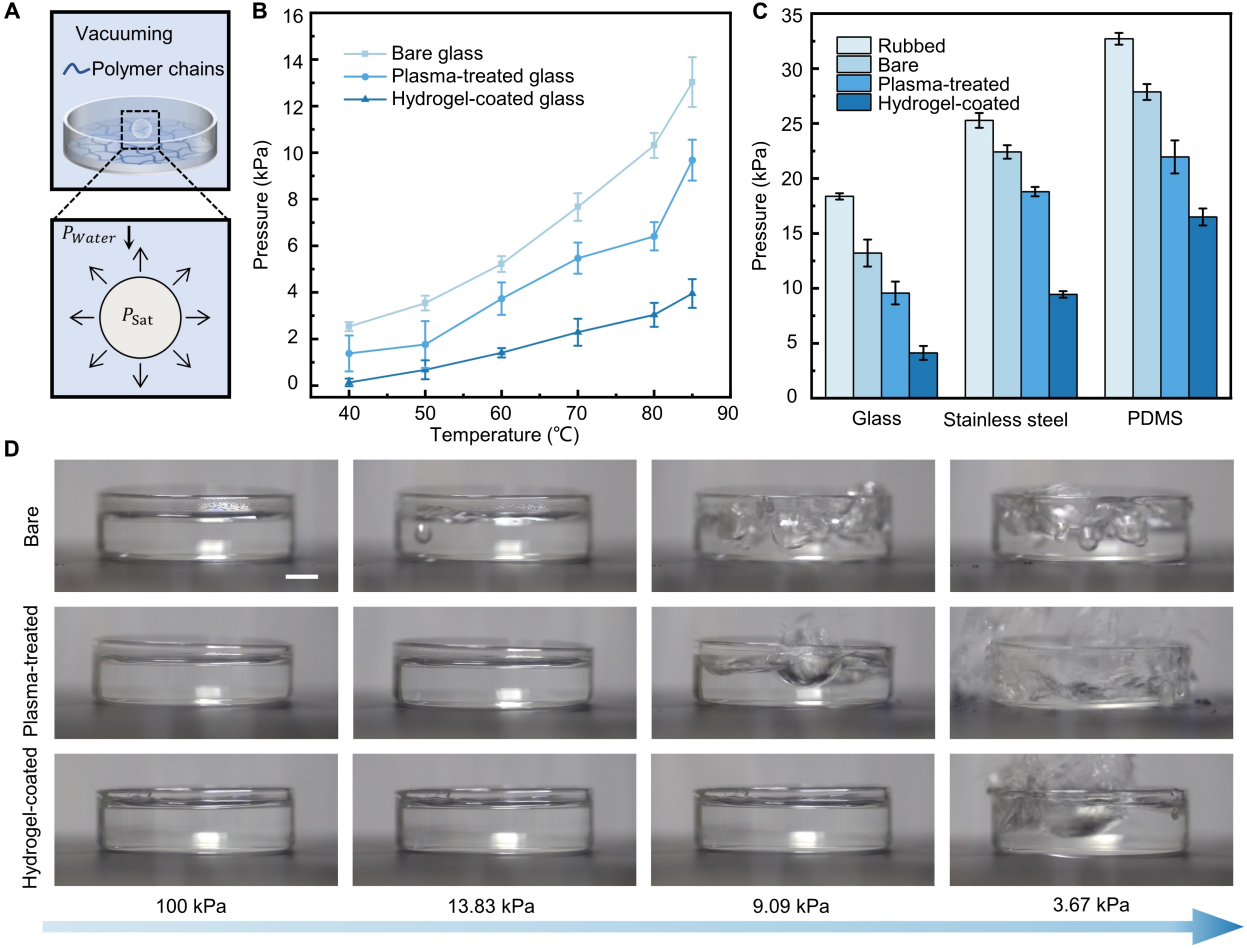
Hydrogel coating inhibits cavitation in evacuation systems. (A) In evacuation experiments, water is contained in a glass petri dish with a hydrogel coating, and a cavitation bubble forms when the external pressure decreases. (B) Plots of the cavitation pressure of water on bare, plasma-treated, and hydrogel-coated glass at different temperatures (40 to 85 °C). (C) Cavitation pressures of water on glass, stainless steel, and polydimethylsiloxane (PDMS) before and after being rubbed, plasma-treated, and hydrogel-coated (85 °C). (D) Experimental snapshots of water cavitation on the surfaces of bare, plasma-treated, and hydrogel-coated glass. The scale bar represents 1 cm.

We performed evacuation experiments to substantiate the inhibitory effect of hydrogel coatings on cavitation (Fig. 2). The cavitation pressure of water at various temperatures (ranging from 40 to 85 °C) was measured in petri dishes composed of bare, plasma-treated, and hydrogel-coated glasses (Fig. 2B). Assuming the generation of a bubble in the water, the internal pressure equals the saturated vapor pressure of water for a given temperature, whereas the external pressure of the liquid water is regarded as the cavitation pressure and detected in the experiment. As the external pressure gradually decreases, the bubble in the water expands spontaneously until the pressure differential is high enough to rupture the water. The glass surface becomes hydrophilic after plasma treatment, resulting in a reduction in cavitation pressure compared to that of bare glass. Furthermore, hydrogel coating inhibits the formation of nuclei. By applying hydrogel coating, the cavitation pressure in a glass petri dish dropped below 5 kPa at all tested temperatures. The hydrogel coating successfully delayed the cavitation phenomenon in pressure dropping experiments. In the case of 85 °C water, cavitation occurred initially on bare glass (13.83 kPa), then on plasma-treated glass (9.09 kPa), and ultimately on hydrogel-coated glass (3.67 kPa) (Fig. 2D and Movie S1). Comparable observations were made on various substrates, including stainless steel and polydimethylsiloxane (Fig. 2C). The influence of surface roughness is validated by the fact that rubbing increases the cavitation pressure on all materials.

We noted the necessity of adhesion between the hydrogel coating and the substrate. The adhesion energy between the glass and the hydrogel coating is comparable to the hydrogel's fracture energies. For comparison purposes, we synthesized bulk hydrogels and attached them to substrates without an adhesion mechanism. In this case, the nucleation energy barrier of the hydrogel–substrate interface is equivalent to that of a water–substrate interface. We observed that as the external pressure decreased, water invaded the hydrogel–substrate interface, and cavitation occurred at the hydrogel–substrate interface at a considerably higher pressure than on adhered hydrogel coatings (Fig. S8 and Movie S2).

Hydrogel coatings demonstrate greater stability over time than hydrophilic treatments. A conventional plasma-treated surface undergoes rapid hydrophobic recovery [35,47], whereas a covalently cross-linked hydrogel is chemically and physically stable [48]. We measured the cavitation pressure on aged plasma-treated and hydrogel-coated glasses. After 72 h, the cavitation pressure of plasma-treated glass was comparable to that of bare glass, whereas hydrogel-coated glass had a lower cavitation pressure (Fig. S9A). Even after 12 d of preservation in water, the hydrogel-coated glass maintained a similar cavitation pressure (Fig. S9B).

**Fig. 3.**
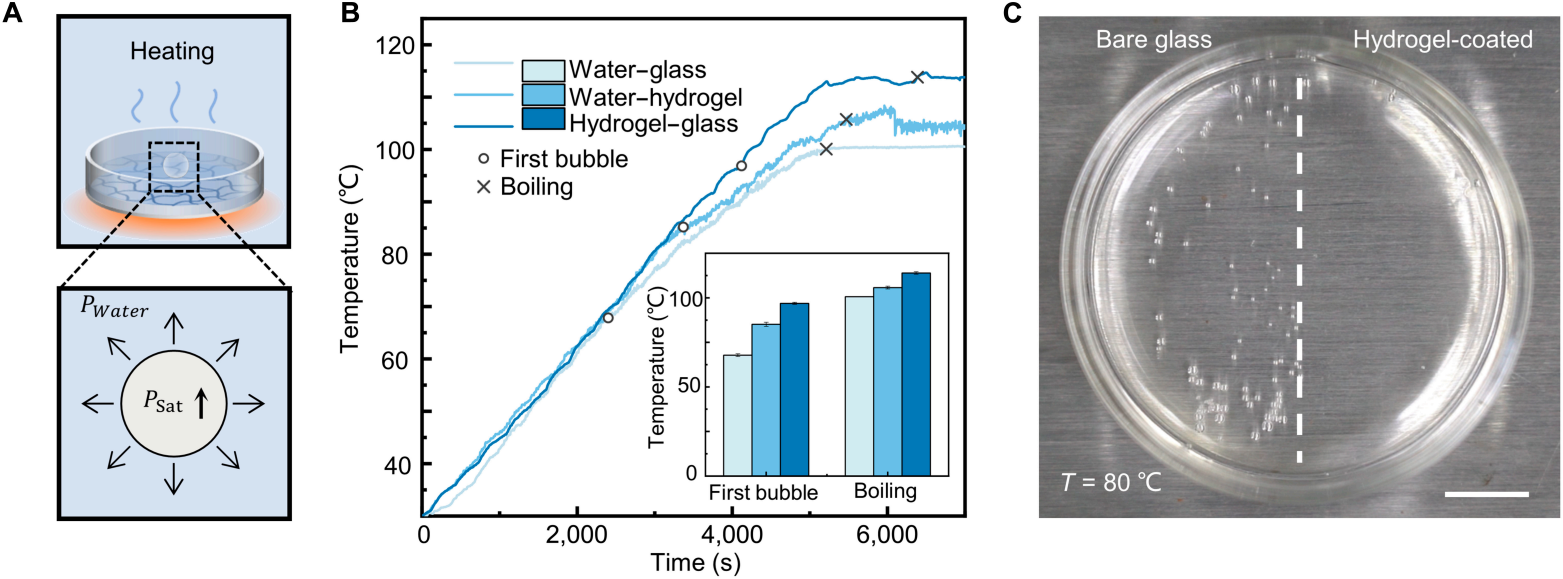
Hydrogel coating inhibits boiling in heating systems. (A) In the heating experiments, water is contained in a glass petri dish with a hydrogel coating, and a boiling bubble forms when the saturated vapor pressure increases with temperature. (B) Temperature profile of water at the water–glass, water–hydrogel, and hydrogel–glass interfaces. The temperature at which the first bubble appears and boiling begins is recorded in the subplot. (C) When water is heated on glass half-coated with hydrogel, boiling mainly occurs at the bare glass surface (left side). The scale bar represents 1 cm.

We conducted heating experiments to validate that hydrogel coatings impeded nucleation during boiling (Fig. 3). For a bubble in water, the inner pressure is equivalent to the saturated vapor pressure of the recorded temperature, whereas the external pressure matches the atmospheric pressure. The pressure differential required for bubble growth is attained by increasing the inner pressure with temperature (Fig. 3A). The temperature of water in both uncoated and hydrogel-coated glass petri dishes was recorded as the temperature gradually increased under atmospheric pressure (Fig. 3B). Our findings indicated that the first bubble formed early at the water–glass interface when the temperature reached 67.82 °C. The bubbles continually carried heat away, and a change in slope was observed in the temperature curve. The temperature was increased by further heating until the water reached the boiling point at 100.60 °C. With the hydrogel coating, the temperature at the hydrogel–glass interface differed from that at the water–hydrogel interface. The temperatures of both interfaces were recorded during heating. At the water–hydrogel interface, bubbles appeared at 85.12 ± 1.17 °C, and the temperature peaked at 108.41 °C, plateauing at around 105.73 ± 0.79 °C. We also observed a gradual rise in temperature at the hydrogel–glass interface until the first interfacial bubble appeared at 96.88 ± 0.55 °C. When the water boiled vigorously, the temperature at the hydrogel–substrate interface remained at approximately 113.91 ± 0.69 °C. Containers with hydrogel coatings required higher temperatures for boiling water compared to uncoated ones. To compare, we used a glass petri dish that was half-coated with hydrogel. When we heated water using this dish, the water on the hydrogel-coated side remained stable while numerous bubbles appeared on the uncoated side. When a boiling bubble appeared at the hydrogel-coated side, intense boiling was observed on the uncoated side (Movie S3).

The nucleation energy barrier, Δ*F*, is overcome by the energy input that correlates with the local heat flow. The water–hydrogel interface has an elevated prefactor *f*(*θ*, *β*, *R*_0_), and the hydrogel–glass interface has a larger interfacial energy *σ*. Their effects on nucleation are comparable. In the evacuation experiments, cavitation occurs solely along the water–hydrogel interface. In the heating experiment, however, during the heat transfer from the bottom of the container to the water, the temperature distribution can lead to an energy variation on the water–hydrogel interface and hydrogel–substrate interface. As the temperature rises, the nucleation energy barrier at the hydrogel–substrate interface can be overcome, resulting in the vulnerability of both the hydrogel–glass interface and the water–hydrogel interface to boiling. We observed that a boiling bubble initiated in a random region along either the water–hydrogel or the hydrogel–substrate interface (Fig. S10). Conversely, a bubble initiated along the water–hydrogel interface grows gradually until it departs by buoyancy. The bubble initiating along the hydrogel–substrate interface grows explosively, rupturing the hydrogel coating and inflating it like a balloon. Following the bubble's penetration of the hydrogel coating, a permanent nucleation site forms. The nucleation temperature at the hydrogel–substrate interface is higher than that at the water–hydrogel interface. As the container thickness increases, both the heating rate of each interface and the peak temperature at the hydrogel–substrate interface decrease during water boiling. However, the peak temperature at the water–hydrogel interface is independent of the container wall thickness (Fig. S11).

### Application for cavitation damage prevention

**Fig. 4.**
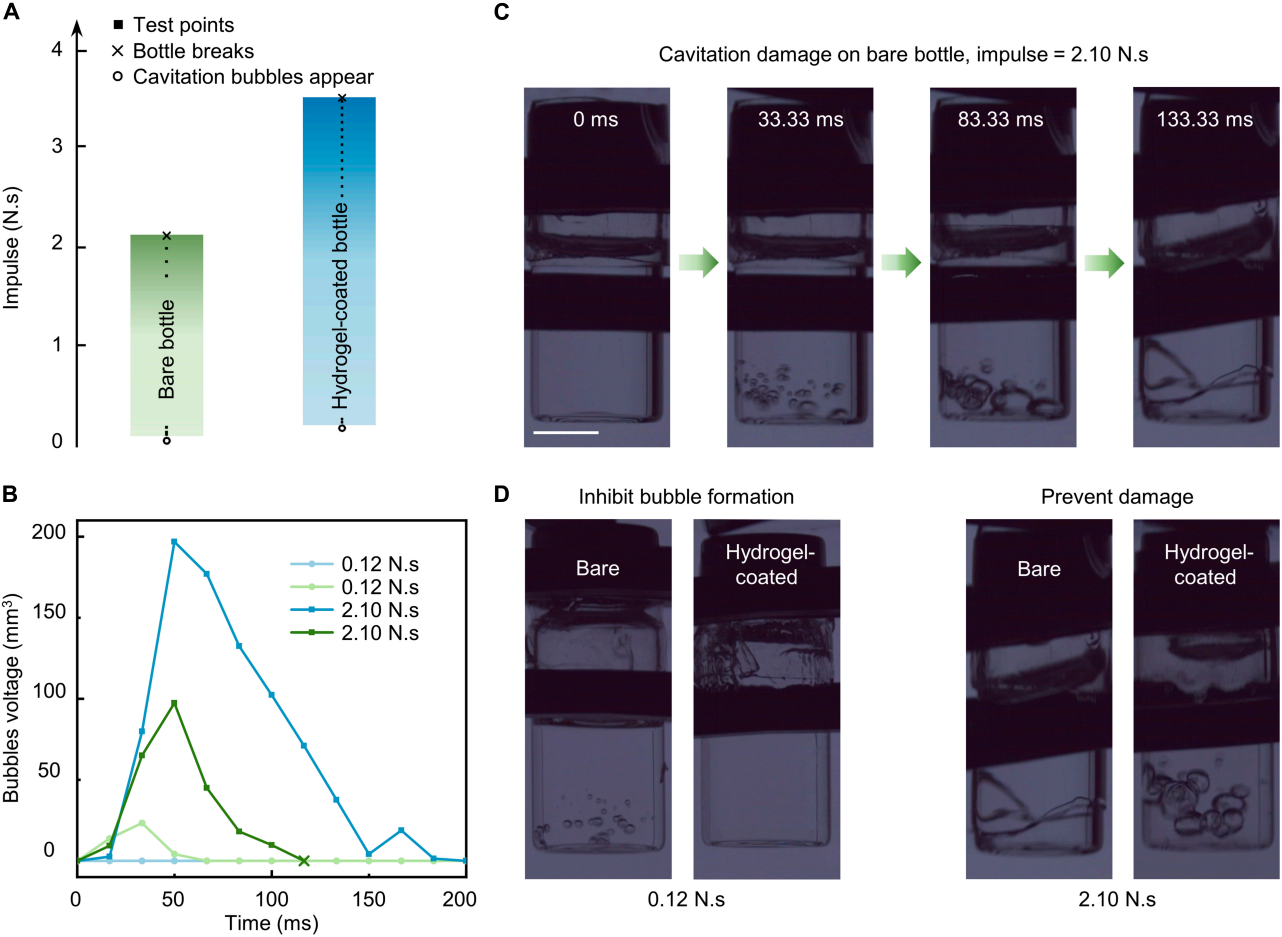
Hydrogel coating prevents damage from acceleration-induced cavitation. (A) Cavitation is detected in bare and hydrogel-coated bottles when they are struck by certain impulses. Solid squares represent test points, hollow circles represent the first detection of cavitation bubbles, while the crosses represent the detection of bottle cracks. (B) Voltage profiles of cavitation bubbles in bare (green lines) and hydrogel-coated (blue lines) bottles struck by 0.12- and 2.10-N.s impulses. (C) Experimental snapshot of a bare bottle struck by 2.10-N.s impulse. (D) Hydrogel coatings inhibit the formation of cavitation bubbles and prevent damage. The scale bar represents 1 cm.

Cavitation occurs in a liquid accelerated by striking. The collapse of cavitation bubbles frequently leads to severe damage [49,50]. For instance, when the top of a water-filled bottle is impacted, the considerable acceleration creates a region of low pressure at the bottom of the bottle where cavitation bubbles form and collapse in an oscillatory event [51,52]. When these cavitation bubbles collapse, the resulting shockwave is typically more potent than the initial impact and causes the bottle to fracture. We illustrate that the application of hydrogel coatings to the inside of a glass bottle can prevent the catastrophic damage resulting from these cavitation events. We struck the hydrogel-coated bottle with a weight, observing the formation, growth, and collapse of cavitation bubbles. After striking, the bottle accelerates, and the water tends to remain in place, causing the liquid pressure at the water–bottle interface to fall below the saturated vapor pressure. Cavitation bubbles form and grow due to the instantaneous negative pressure. After acceleration, the pressure in the water returns to normal, and the bubble collapses in an oscillatory fashion. Coating with hydrogels would elevate the energy barrier for cavity nucleation. As compared to bare bottles, greater impulse (elevated from 0.12 to 0.19 N.s) is required to generate detectable cavitation bubbles on hydrogel-coated bottles. The hydrogel coating can inhibit cavitation at the bottom of the bottle during accelerated impact. In addition, a bare bottle cracks with a 2.10-N.s impulse, but a hydrogel-coated bottle can resist a 3.53-N.s impulse before cracking (Fig. 4 and Movies S5 and S6). The main mechanism of cavitation inhibition by hydrogel coatings is to increase the energy barrier for nucleation, reducing the generation of cavitation bubbles, thereby decreasing the energy of bubble collapse and weakening the impact damage of cavitation bubbles on the vessel.

## Discussion

To summarize, we have demonstrated that hydrogel coatings can inhibit heterogeneous nucleation in water. We have derived that the resilience, smoothness, and hydrophilicity of the hydrogel coating [52] elevate the energy barrier for heterogeneous nucleation and raise the phase transition limit of water in practical use. Furthermore, we have conducted of All-Atom Molecular Dynamics (AAMD) simulations to verify that nucleation of water is confined within hydrogel coatings that consist of a hydrophilic polymer network. Our results indicate that hydrogel coatings reduce the pressure threshold for heterogeneous cavitation and elevate the superheating temperature of water prior to boiling. Moreover, hydrogel coatings can avert cavitation damage in accelerating systems and show great potential in preventing explosive boiling in heat transfer and cavitation erosion in fluidic systems. Analogous effects may also be observed in other solvents when hydrogels swell to their equilibrium state in corresponding liquids.

## Materials and Methods

### Preparation of hydrogel coating

To produce the hydrogel precursor, AAm (AAm, Aladdin, A1084ff65) was dissolved in deionized water to form a 2 M solution. For every 1 ml of the solution, 20 μl of 0.1 M α-ketoglutaric acid (Sigma-Aldrich, 75890) was added as a photoinitiator, 1.9 μl of 3-(trimethoxysilyl)propyl methacrylate (Sigma-Aldrich, 440159) was added as a cross-linker, and 5.5 μl of (3-mercaptopropyl) trimethoxysilane (Sigma-Aldrich, 175617, 0.1% volume ratio in tetrahydrofuran) was added as a chain transfer agent. To disperse, hydrolyze, and dissolve 3-(trimethoxysilyl)propyl methacrylate, the solution was stirred for 60 s. Then, the solution was preserved in syringes and ultrasonically degassed to create an anaerobic environment, allowing it to polymerize in 25 min under ultraviolet light (365 nm, 9,999 mJ cm^−2^ power). The substrates were pre-treated in oxygen plasma for 2 min. The precursor was then slowly applied to the substrates to create a uniform coating, which was then cured at 65 °C for 12 h. Finally, the as-prepared hydrogel coating swelled to an equilibrium state in pure water for 48 h. Unless otherwise specified, the fully swelled hydrogel coating was used for subsequent experiments.

To prepare bulk hydrogel, for every 1 ml of the 2 M AAm (Aladdin, A108465) solution, 20 μl of 0.1 M α-ketoglutaric acid (Sigma-Aldrich, 75890) was added as a photoinitiator, 40 μl of 0.1 M N,N′-methylenebisacrylamide (Sigma-Aldrich, 101546) was added as a cross-linker, and 5.5 μl of (3-mercaptopropyl) trimethoxysilane (Sigma-Aldrich, 175617, 0.1% volume ratio in tetrahydrofuran) was added as a chain transfer agent. The solution was then exposed to ultraviolet radiation for 30 min to create bulk hydrogel.

### Evacuation experiment

The apparatus utilized for the evacuation experiment primarily consisted of a drying dish equipped with a digital pressure gauge, with a range spanning from −100 to 0 kPa, as well as a vacuum pump. The drying dish and vacuum pump were linked through an 8-mm-diameter polyvinyl chloride hose. We heated water to a desired temperature of either 40, 50, 60, 70, 80, or 85 °C. The water used for the experiments was deionized and defoamed by ultrasound. Temperatures below 40 °C were avoided as water was less prone to cavitation at such temperatures. In addition, we preheated the bare, plasma-treated, and hydrogel-coated glass dishes and added 10 ml of heated water to each. We then initiated the vacuum pump and noted the cavitation pressure of water.

### Heating experiment

When heating water in glass dishes with or without hydrogel coating, the heat was transferred from the hot table to the water, creating a temperature gradient. Ultrathin thermocouples, measuring 0.1 mm in diameter and of Type K, were utilized to measure the temperature of water at the hydrogel–substrate and water–hydrogel interfaces. The thermocouples were coated with hydrogel and were connected to a thermometer, which recorded the water temperature in real time. Deionized water (20 ml) at 30 °C was added to the glass dishes, and the temperature of the hot table was increased by 10 °C every 5 min until the water had boiled.

### Mechanical tests

The adhesion energy at the hydrogel–glass interface and the fracture energy of hydrogel were measured using a standard 90° peeling test (Instron 5465). The peeling rate was 10 mm min^−1^. To test the adhesion energy, a hydrogel with dimensions of 70 mm × 20 mm × 4.5 mm was painted on bare glass. A poly(methylmethacrylate) film (thickness = 70 μm; Anyuan tech) was then affixed to the sample as a backing layer using instant adhesive (PR100, 3 M) (Fig. S6A). A 2-cm precrack was introduced at the hydrogel–glass interface. To test the fracture energy of hydrogel, both sides of the hydrogel were bonded to the backing layers, and a precrack was introduced in the center of the hydrogel. The samples were loaded to Instron with a 10 N load cell. As the system moved at a constant speed, the force signal displayed a transient regime followed by a smooth state where the force *F* was measured. The 90° peeling energy *G* (N m^–1^) was given by: *G* = *F*/*l*, where *l* was the width of hydrogel coating bonded to the backing layer. The polymer network stretched upon swelling, which led to a decrease in polymer chain density, and consequently a reduction in fracture energy (Fig. 1D). Cohesive failure occurred during the adhesion energy test. Although the adhesion energy was found to be similar to the fracture energy of the hydrogel, the interfacial energy was expected to be greater. The data verified that the lower limit of nucleation energy barrier could be elevated by hydrogel coating, and thus, heterogeneous nucleation could be inhibited.

### Contact angle

We deposited 7 μl of water on 4 different surfaces: bare glass, plasma-treated glass, as-prepared hydrogel, and swelled hydrogel (70 mm × 20 mm × 1 mm). Using a macro camera, we captured images of the water droplets on each surface and analyzed the contact angle of the water.

### Surface roughness

The surface roughness of the as-prepared hydrogel, the swelled hydrogel, the rubbed hydrogel, the glass, and the rubbed glass were measured by a 3-dimensional interference microscope (Veeco; NT9100).

### Acceleration-induced cavitation

We struck glass bottles (containing 5- and 8-ml water) with weights (50 and 500 g) at various heights (20 to 260 cm) and recorded the formation, growth, and collapse of cavitation bubbles using a high-speed camera (Chronos 2.1-Kron).

### The AAMD simulation

The AAMD simulations were conducted using a large-scale atomic/molecular massively parallel simulator and a consistent valence force field [53,54]. A Nose–Hoover thermostat was used to regulate the system temperature [55,56]. For the water–silicon system, a 3-dimensional homogeneous component sample of silicon with a size of 10.5 × 10.5 × 3 nm^3^ was created and then combined with a water component sample of the same dimensions containing ~30,000 atoms. The simulated hydrogel system consisted of 90% water (~6,000 atoms) and 10% polyacrylamide, which contained 12 individual chains, each with 40 monomers. The system had dimensions of 11 × 11 × 10 nm^3^. The 2 systems were initially equilibrated at 300 K for 5 × 10^6^ timesteps (dt = 1 fs) using NPT dynamics, followed by uniaxial loading along the Z-axis at a constant stress rate of 5 × 10^12^ MPa s^−1^. Periodic boundary conditions were applied in all 3 directions throughout the simulation, with constant temperature (300 K) and zero pressure in the nonstretching directions (i.e., X- and Y-axes). Molecular visualization and nucleation analysis were carried out using the visual molecular dynamics graphics software.

### Continuous rub test

The rub test was conducted in water using a rheometer (Anton-Paar, MCR302). The hydrogel coating was adhered to a glass dish (60-mm diameter). A load cell (parallel plate, 25-mm diameter) was applied on the surface of the hydrogel with an initial normal stress of 400 Pa and rotated in one direction for 3 h at an angular velocity of 1 rad s^−1^. The friction coefficient was obtained by the relation of *μ* = 4*T*/3*RF*, where *R* was the diameter of the load cell, *T* was the torque, and *F* was the normal force.

## Data Availability

All data are available in the main text or the Supplementary Materials.
