# Supplementary material for: Inhibition of Heterogeneous Nucleation in Water by Hydrogel Coating
Source: Research (Wash D C). 2023 Jul 6;6:0190. doi: 10.34133/research.0190 (PMC10325670; doi:10.34133/research.0190)
Supplement: Supplementary 1 — Figs. S1 to S12 Movies S1 to S7 [file research.0190.f1.zip › SI.docx]

Supplemental Material for

**Inhibition of heterogeneous nucleation in water by hydrogel coating**

Siyang Li^1,2,3^, Panpan Zhu^1,2,3^, Yaoting Xue^1,2,3^, Lei Wang^1,2,3^, Tuck-Whye Wong^1,2,3,4^, Xuxu Yang^1,2,3*^, Haofei Zhou^1,2,3^, Tiefeng Li^1,2,3^, Wei Yang^1,2,3^

Affiliations:

^1^ Department of Engineering Mechanics, Zhejiang University, Hangzhou 310027, China.

^2^ Key Laboratory of Soft Machines and Smart Devices of Zhejiang Province, Zhejiang University, Hangzhou 310027, China.

^3^ Center for X-Mechanics, Department of Engineering Mechanics, Zhejiang University, Hangzhou 310027, China.

^4^ School of Biomedical Engineering and Health Sciences and Advanced Membrane Technology Research Centre, Universiti Teknologi Malaysia, Skudai, 81310, Malaysia.

* Email: xxyang@zju.edu.cn (Xuxu Yang).

**This PDF file includes：**

Supplementary Figures S1 to S12

Supplementary Movie Captions

**Other Supplementary Materials for this manuscript include:**

Supplementary Movies S1 to S7

**Nucleation energy barrier**

In the process of homogeneous nucleation, the critical nuclei are assumed to be in thermodynamic equilibrium with their surroundings. According to the classical nucleation theory [1-4], the energy required to create a vapor of radius *R* in water is

$$\begin{aligned} \Delta F=4\pi R^{2}\sigma-\frac{4}{3}\pi R^{3}\left( p_{sat}-p_{liq} \right),\#\left( 1 \right) \end{aligned}$$

where the first term accounts for the energy gained through the creation of a void in liquid water, whose surface energy per unit area is *σ*, and the second term accounts for the energy lost through the formation of a bubble in the liquid phase. *p*_sat_ is the saturated vapor pressure of water at the current temperature, and *p*_liq_ is the pressure of liquid water which follows the Clapeyron equation [5]:

$$\begin{aligned} lnp_{sat}=-\frac{\Delta H_{vap}}{RT}+C,\#\left( 2 \right) \end{aligned}$$

where $\Delta H_{vap}$ is the molar enthalpy of vaporization, *R* is the gas constant (8.314 J mol^-1^K^-1^), *T* is the absolute temperature, and *C* is a constant of integration. The required energy rises first with vapor radius until reaching an energy barrier at a critical radius *R*_c_, and then declines. At the critical radius

$$\begin{aligned} R_{C}=\frac{2\sigma}{p_{sat}-p_{liq}},\#\left( 3 \right) \end{aligned}$$

the energy barrier reaches

$$\begin{aligned} {\Delta F}_{max}=\frac{16\pi\sigma^{3}}{{3\left( p_{sat}-p_{liq} \right)}^{2}}=\frac{4}{3}\pi{R_{C}}^{2}\sigma,\#\left( 4 \right) \end{aligned}$$

and the vapor bubble will grow spontaneously after the energy barrier is overcome. This energy barrier applies when nucleation occurs homogeneously in water.

In practice, nucleation occurs heterogeneously along the interface between water and solids. In the theory of heterogeneous nucleation, when the solid surface is defect free, the volume of the bubble is

$$\begin{aligned} V=\frac{1}{3}\pi R^{3}{(1+cos\theta)}^{2}\left( 2-cos\theta\right),\#\left( 5 \right) \end{aligned}$$

where $\theta$ is the contact angle of water on the solid. The energy required to create a vapor becomes

$$\begin{aligned} \Delta F=\left[ 4\pi R^{2}\sigma-\frac{4}{3}\pi R^{3}\left| \delta P \right| \right]\frac{{(1+cos\theta)}^{2}(2-cos\theta)}{4}，\#\left( 6 \right) \end{aligned}$$

where $\delta P=p_{sat}-p_{liq}$. At a critical radius of $R_{C}=2\sigma/\delta P$, the energy barrier can be evaluated as

$$\begin{aligned} {\Delta F}_{max}=\frac{4}{3}\pi{(1+cos\theta)}^{2}\left( 2-cos\theta\right)\frac{\sigma^{3}}{\left| \delta P \right|^{2}}=\frac{1}{3}\pi{R_{c}}^{2}\sigma\left( 1+cos\theta\right)^{2}\left( 2-cos\theta\right).\#\left( 7 \right) \end{aligned}$$

When the water wets the substrate completely, we have $\theta=0$, and $\left( 1+cos\theta\right)^{2}\left( 2-cos\theta\right)=4$. The heterogeneous energy barrier in equation (7) is equal to the homogeneous energy barrier in equation (4). Incomplete wetting results in a reduction in the energy barrier.

When the solid surface is rough, the microscopic geometry of the surface is considered as cones with semi-angle of *β*. Then the energy barrier becomes

$$\begin{aligned} {\Delta F}_{max}=\left[ 4\pi R^{2}\sigma-\frac{4}{3}\pi R^{3}\left| \delta P \right| \right]f\left( \theta,\beta\right)=\left( \frac{4}{3}\pi{R_{C}}^{2}\sigma\right)f\left( \theta,\beta\right),\#\left( 8 \right) \end{aligned}$$

where

$$\begin{aligned} f\left( \theta,\beta\right)=\frac{1}{2}-\frac{\sin\left( \theta-\beta\right)}{2}+\frac{{cos}^{2}(\theta-\beta)cos\theta}{4sin\beta}.\#\left( 9 \right) \end{aligned}$$

When a small bubble with a radius of *R_0_* is gaped at the interface, the energy required for nucleation is reduced to

$$\begin{aligned} {\Delta F}_{max}=\left( \frac{4}{3}\pi{R_{C}}^{2}\sigma\right)f\left( \theta,\beta\right)-\left( \frac{4}{3}\pi{R_{0}}^{2}\sigma\right)f\left( \theta,\beta\right)=\left( \frac{4}{3}\pi{R_{C}}^{2}\sigma\right)f\left( \theta,\beta,R_{0} \right),\#\left( 10 \right) \end{aligned}$$

where

$$\begin{aligned} f\left( \theta,\beta,R_{0} \right)=\left( \frac{1}{2}-\frac{\sin\left( \theta-\beta\right)}{2}+\frac{{cos}^{2}(\theta-\beta)cos\theta}{4sin\beta} \right)\left( 1-\frac{{R_{0}}^{2}}{{R_{C}}^{2}} \right)\#\left( 11 \right) \end{aligned}$$

stands for the prefactor of the energy required for nucleation when the nucleation is heterogeneous.

In the experiment, water can reach metastable states before nucleation through two ways: by being stretched below its saturated vapor pressure or superheated above its boiling temperature. The nucleation rate can be approximated using the thin wall model as a function of temperature and pressure:

$$\begin{aligned} \Gamma=\Gamma_{0}exp \left( -\frac{{\Delta F}_{max}}{kT} \right),\#\left( 12 \right) \end{aligned}$$

where *k* is Boltzmann’s constant, and the prefactor

$$\begin{aligned} \Gamma_{0}\cong\left( \frac{4}{3}\pi R_{c}^{3} \right)^{-1}\frac{kT}{h},\#\left( 13 \right) \end{aligned}$$

is the product of the number of nucleation sites per unit volume and the frequency of nucleation attempts per unit time. *h* is Planck’s constant.

The probability of nucleation occurring in time *τ* for a sample of volume *V* is given by

$$\begin{aligned} S=1-exp \left( -\Gamma V\tau\right).\#\left( 14 \right) \end{aligned}$$

If the probability of cavitation $S=1/2$, we have

$$\begin{aligned} {\Delta F}_{max}=kTln\left( \frac{\Gamma_{0}V\tau}{ln2} \right).\#(15) \end{aligned}$$

Based on equation (10) and (15), $\delta P$ can be written as

$$\begin{aligned} \left| \delta P \right|=\sqrt{\frac{16\pi\sigma^{3}f\left( \theta,\beta,R_{0} \right)}{3kTln\left( \frac{\Gamma_{0}V\tau}{ln2} \right)}}.\#(16) \end{aligned}$$

As illustrated in section A, a hydrogel coating alters the energy landscape of nucleation in a water-containing system by increasing the surface energy, *σ*, and the prefactor, $f\left( \theta,\beta,R_{0} \right)$. Following equation (16), when a hydrogel coating is applied to the solid-water interface, the required pressure difference between the saturated vapor pressure and the liquid pressure, $\delta P=p_{sat}-p_{liq}$, should be increased to reach a certain nucleation rate. If the temperature is fixed, $p_{sat}$ remains constant, and an increased $\delta P$ means that the water can resist a lower $p_{liq}$. If the water is kept at atmospheric pressure, $p_{liq}$ remains constant, and a high temperature is required to increase $\delta P$ for boiling water, following the Clapeyron equation. We conducted evacuation and boiling experiments to validate this theory.


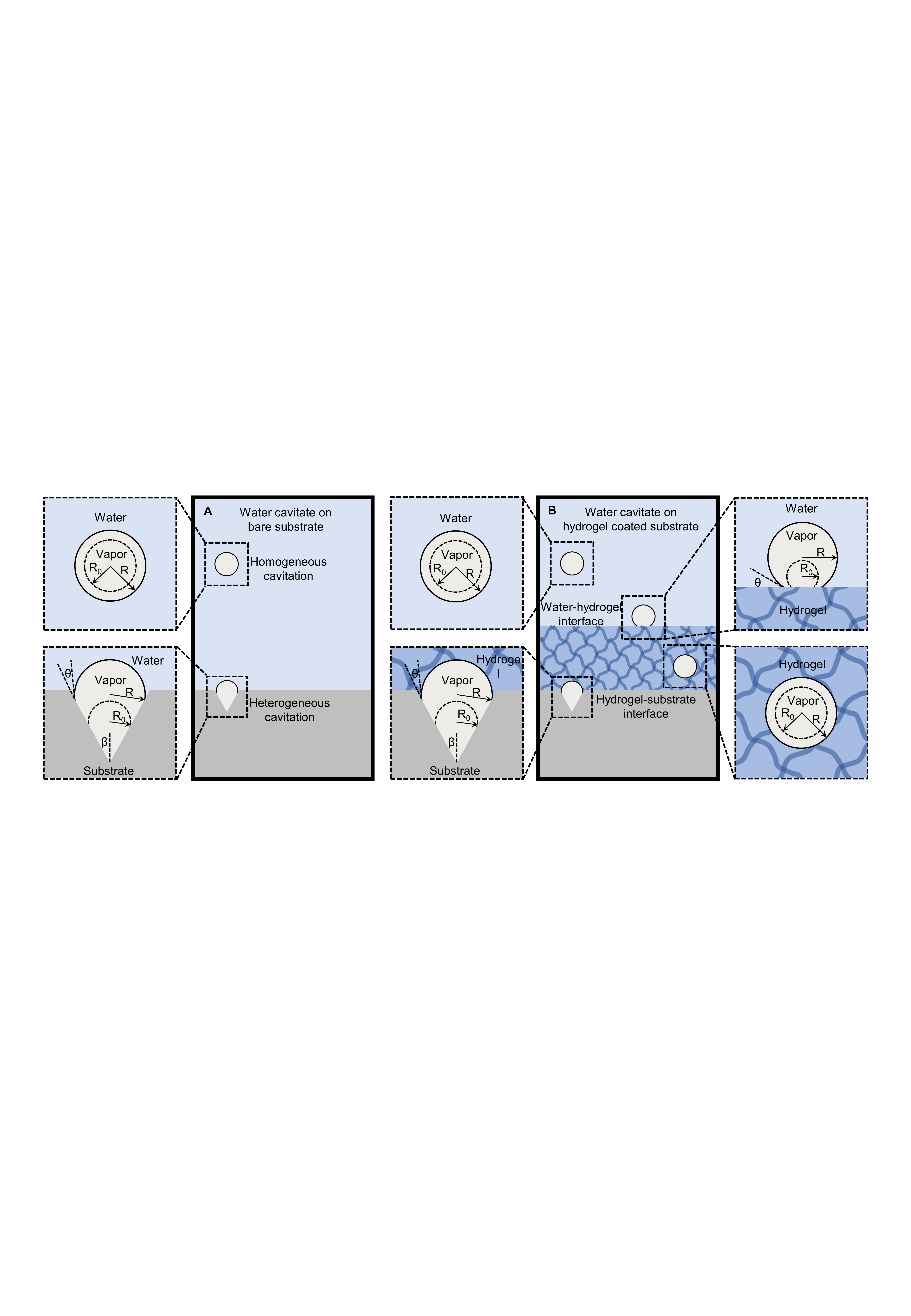


Fig. S1. Schematic illustration of the cavitation of water in regions with and without hydrogel coating. (A) On a bare substrate, nucleation may occur in water, or along the water-substrate interface. (B) On a hydrogel coated substrate, nucleation may occur in water, along the water-hydrogel interface, in hydrogel, or along the hydrogel-substrate interface.


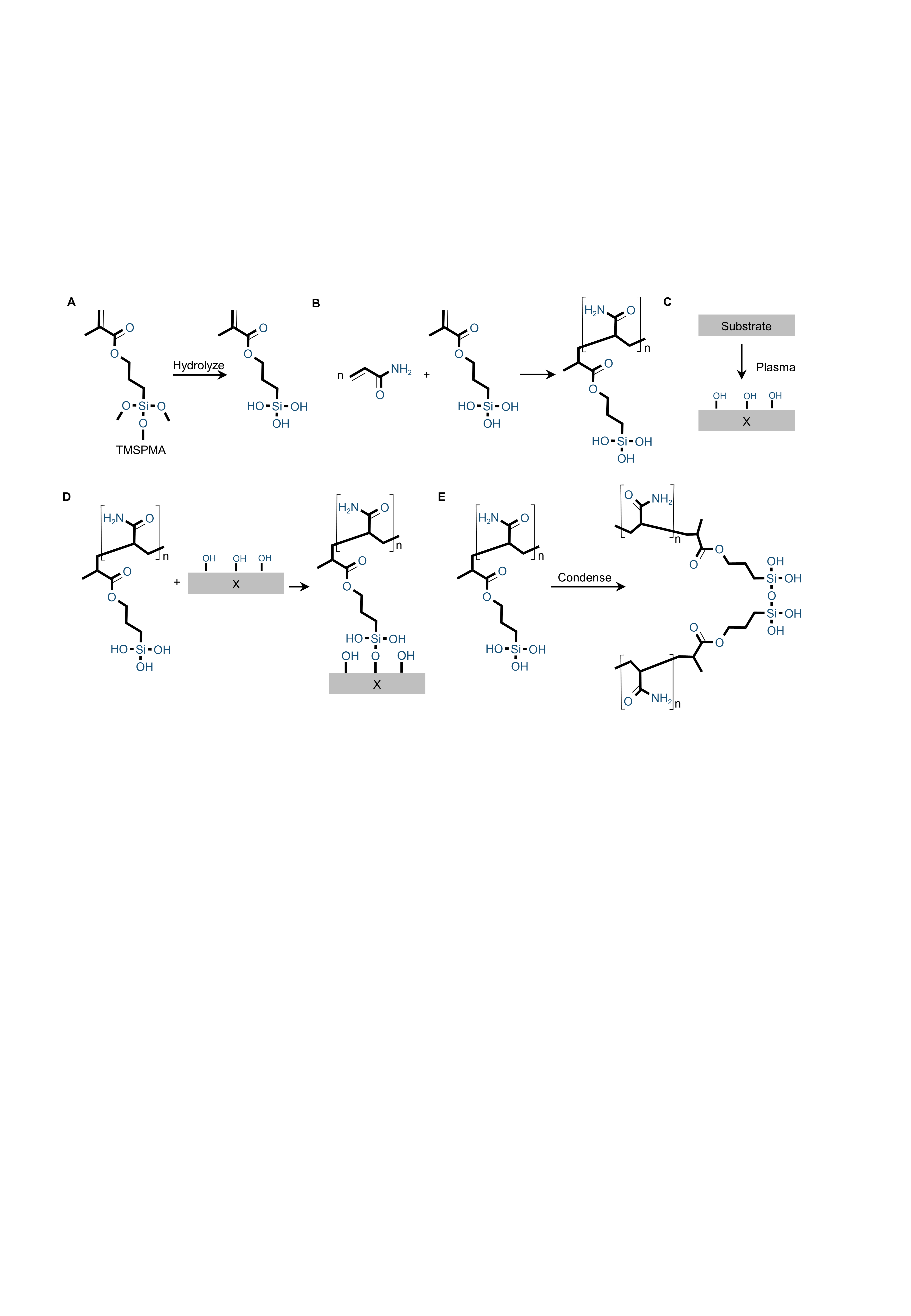


Fig. S2. Schematic illustration of the chemistry of anchoring hydrogel networks to solid surfaces.


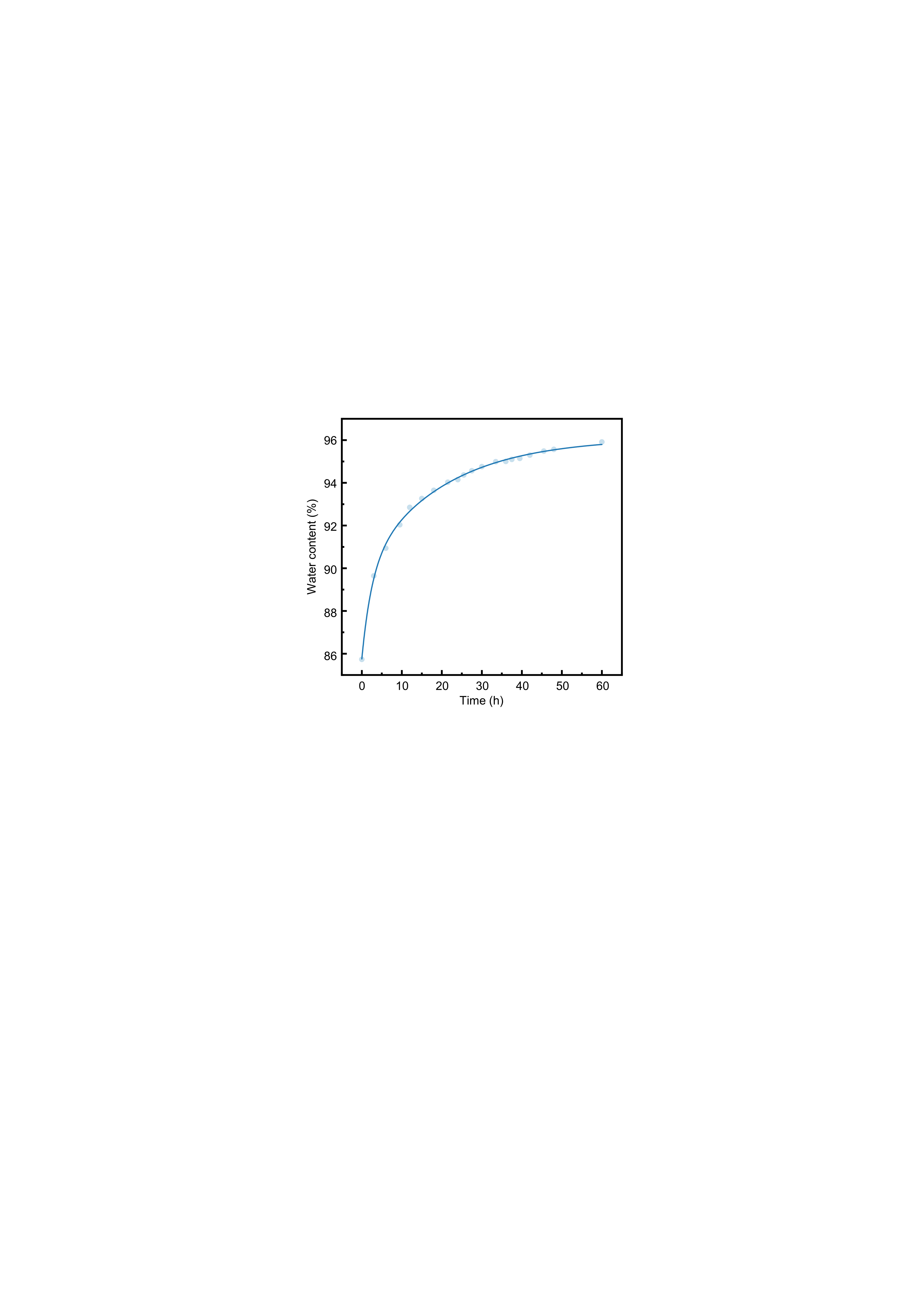


Fig. S3. Water content of the hydrogel coating when immersed in water.


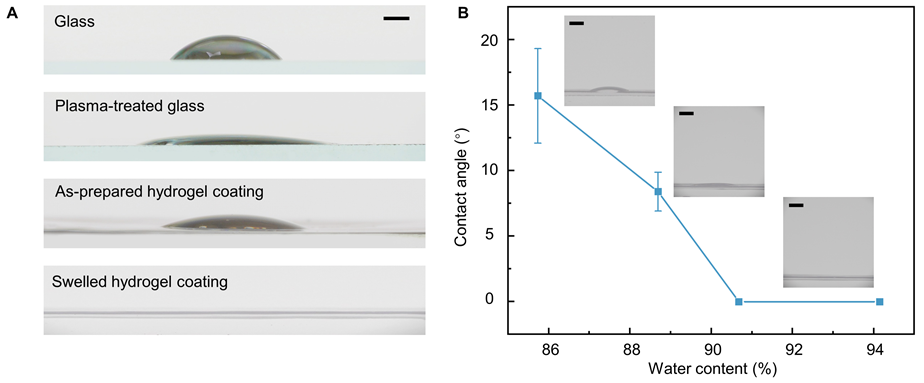


Fig. S4. (A) Contact angles of water on bare, plasma-treated, as-prepared hydrogel-coated, and fully swelled hydrogel-coated glass. The Scale bar represents 1 mm. (B) Contact angles of water on hydrogels with different water contents. The Scale bars represent 2 mm.


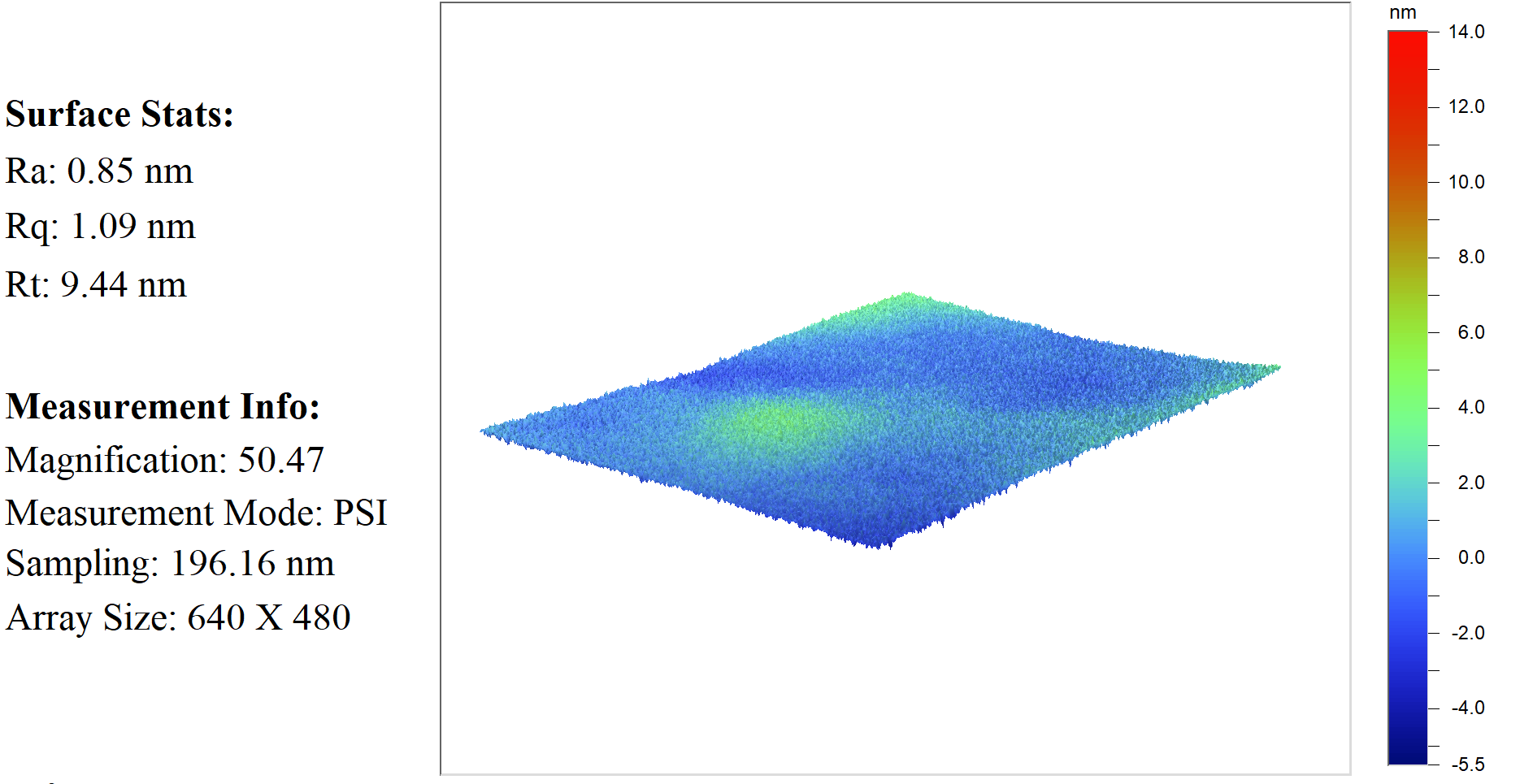


Fig. S5. Three-dimensional surface of as-prepared hydrogel coating obtained by a three-dimensional interference microscope (Veeco, NT9100).


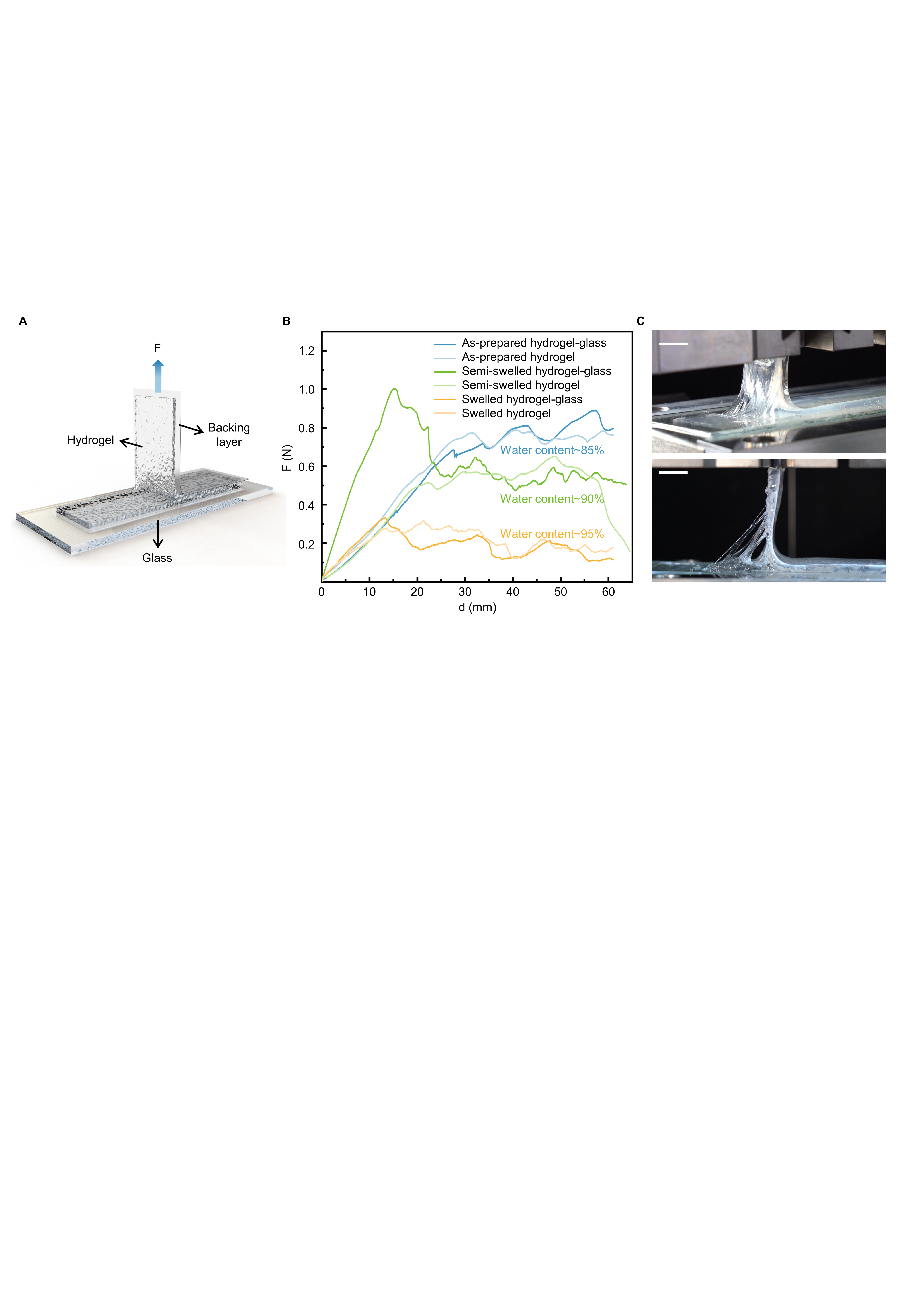


Fig. S6. Fracture energy test of the hydrogel and adhesion energy test of the hydrogel-glass interface. (A) The experiment setup for 90°peeling test. (B) The force-displacement curves of hydrogels with different water contents. Due to cohesive failure, the fracture energies of the hydrogels and the adhesion energies of the hydrogel-glass interfaces were comparable. (C) During the 90° peeling experiments, cohesive failure was observed. The sample width is 2 cm. The Scale bars represent 1 cm.


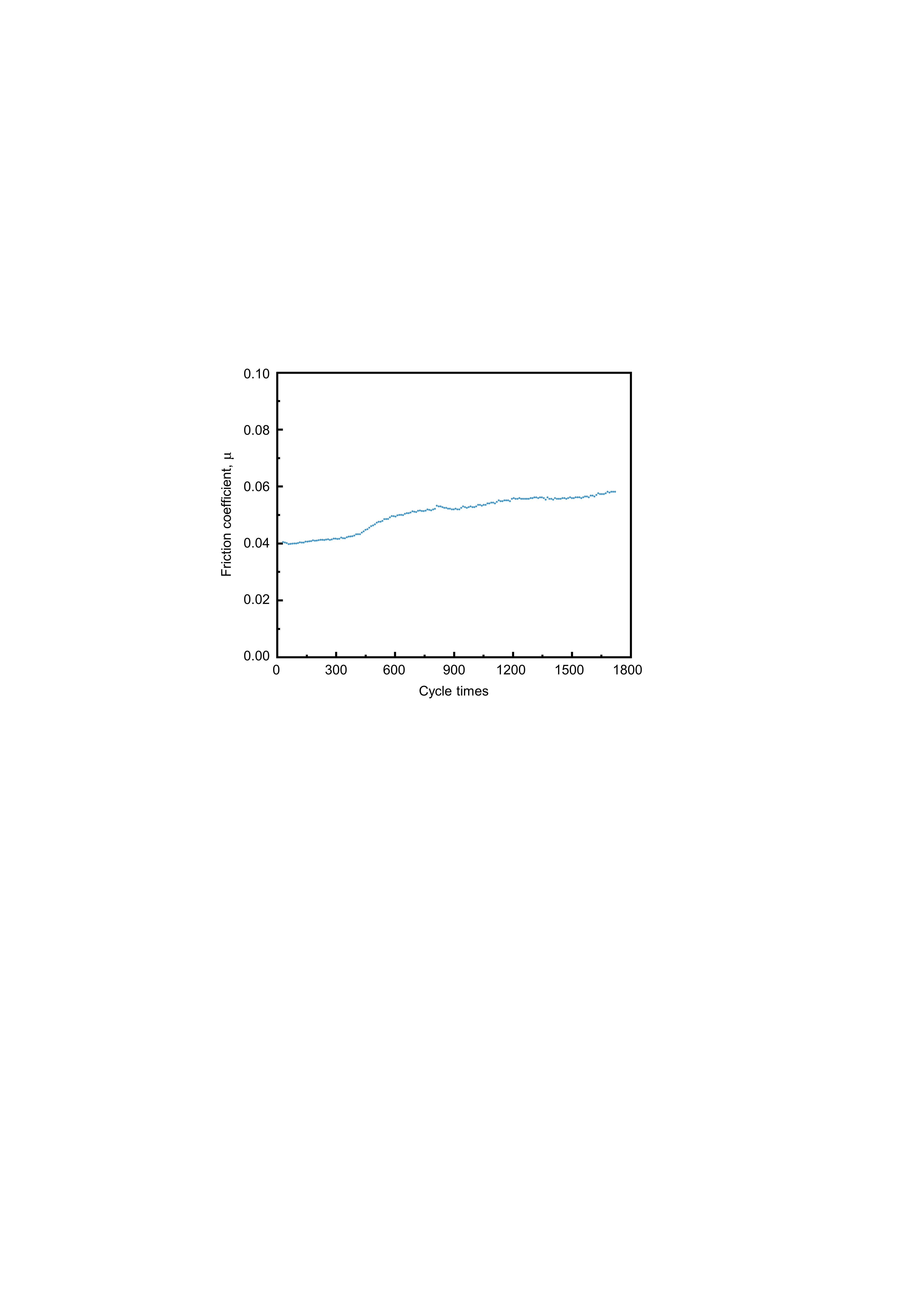


Fig. S7. The friction coefficient of the hydrogel coating during a continuous rub test by a rheometer.


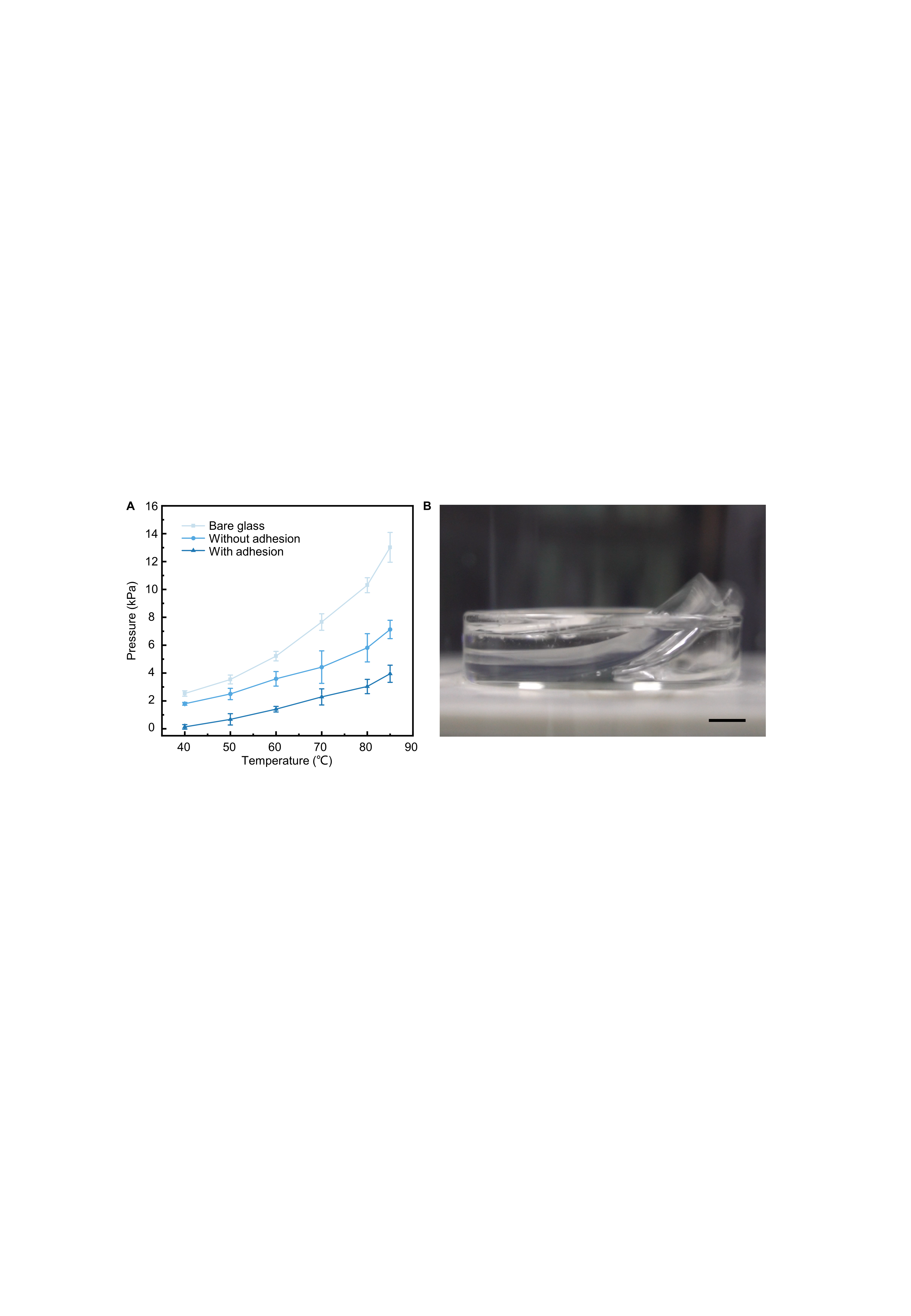


Fig. S8. Influence of hydrogel-glass interfacial adhesion on cavitation pressure. (A) In various temperatures, the cavitation pressure of water on bare glass, and on hydrogel that is or is not adhered to the glass. (B) When the hydrogel is not adhered to the glass, cavitation occurs along the hydrogel-glass interface. The cavitation pressure is slightly reduced by bulk hydrogel on the substrate without adhesion, the weight of the hydrogel resists the expansion of the bubbles. The Scale bar represents 1 cm.


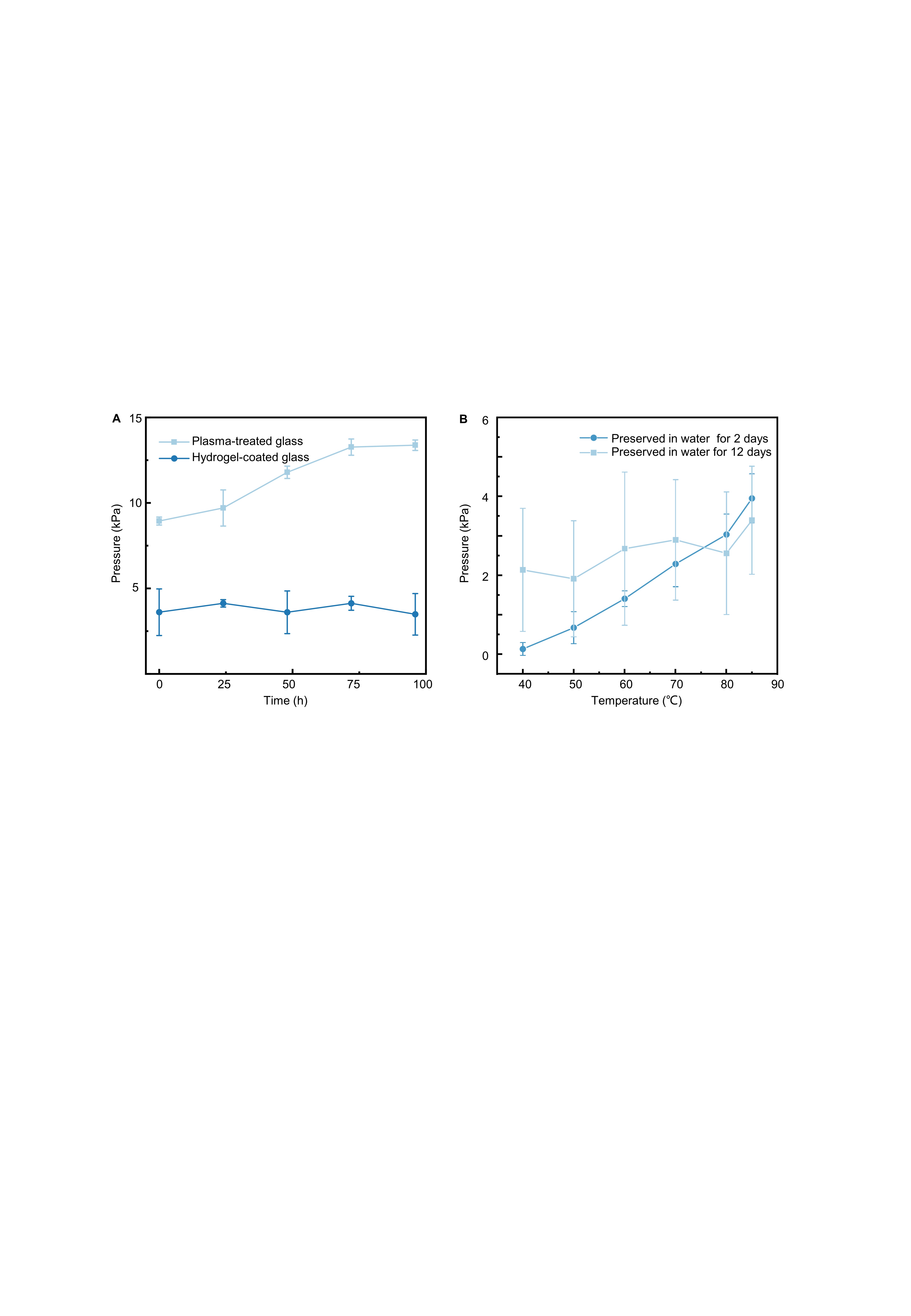


Fig. S9. (A) Cavitation pressure of 85 °C water on plasma-treated and hydrogel-coated glass after they are prepared and preserved in water for a time period. (B) Cavitation pressure of water on hydrogel-coated glass at different temperatures (40 °C-85 °C), after the coatings are prepared and preserved in water for 2 and 12 days.


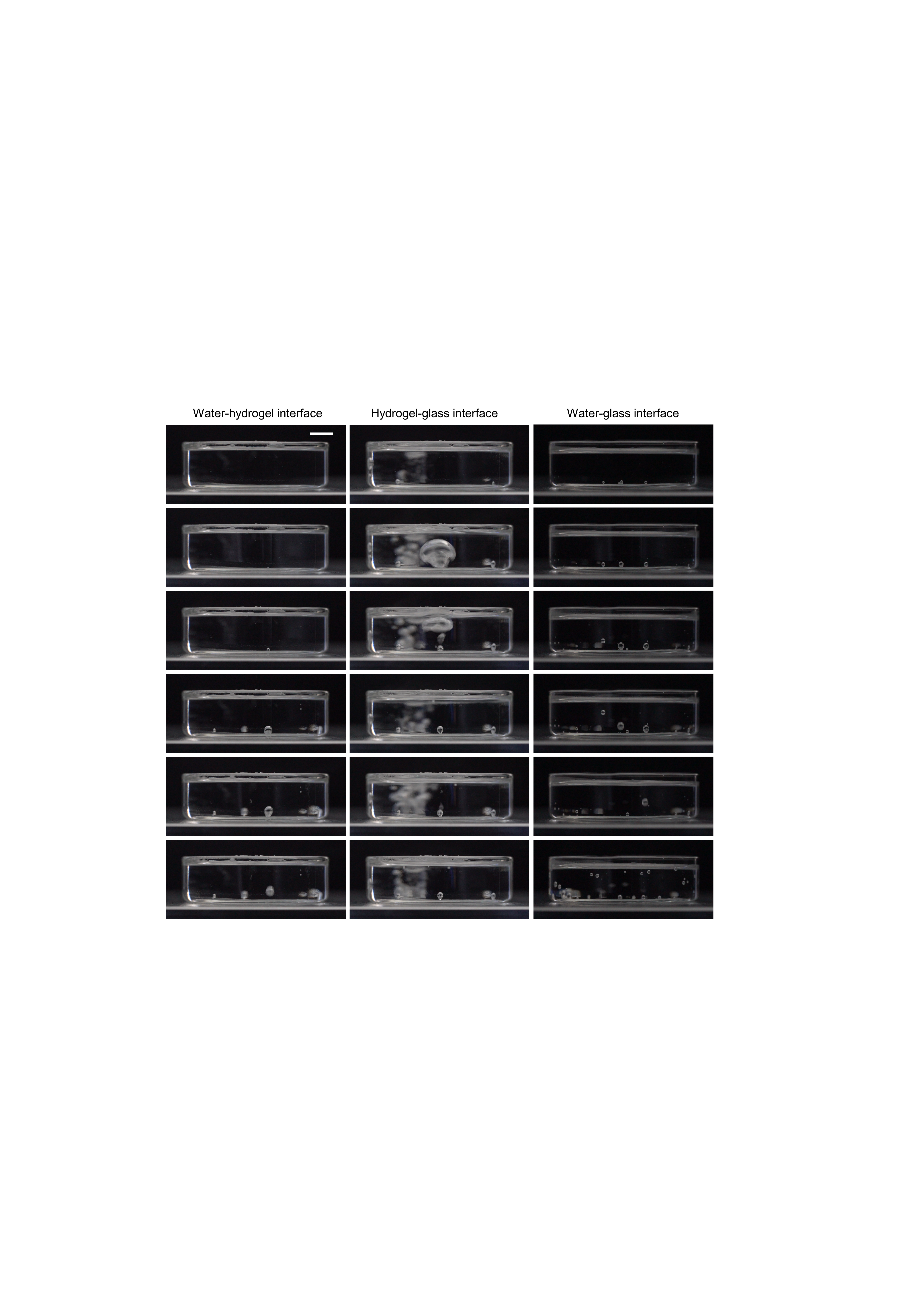


Fig. S10. Experimental snapshots of boiling bubbles along the water-hydrogel, the hydrogel-glass and the water-glass interfaces. The Scale bar represents 1 cm.


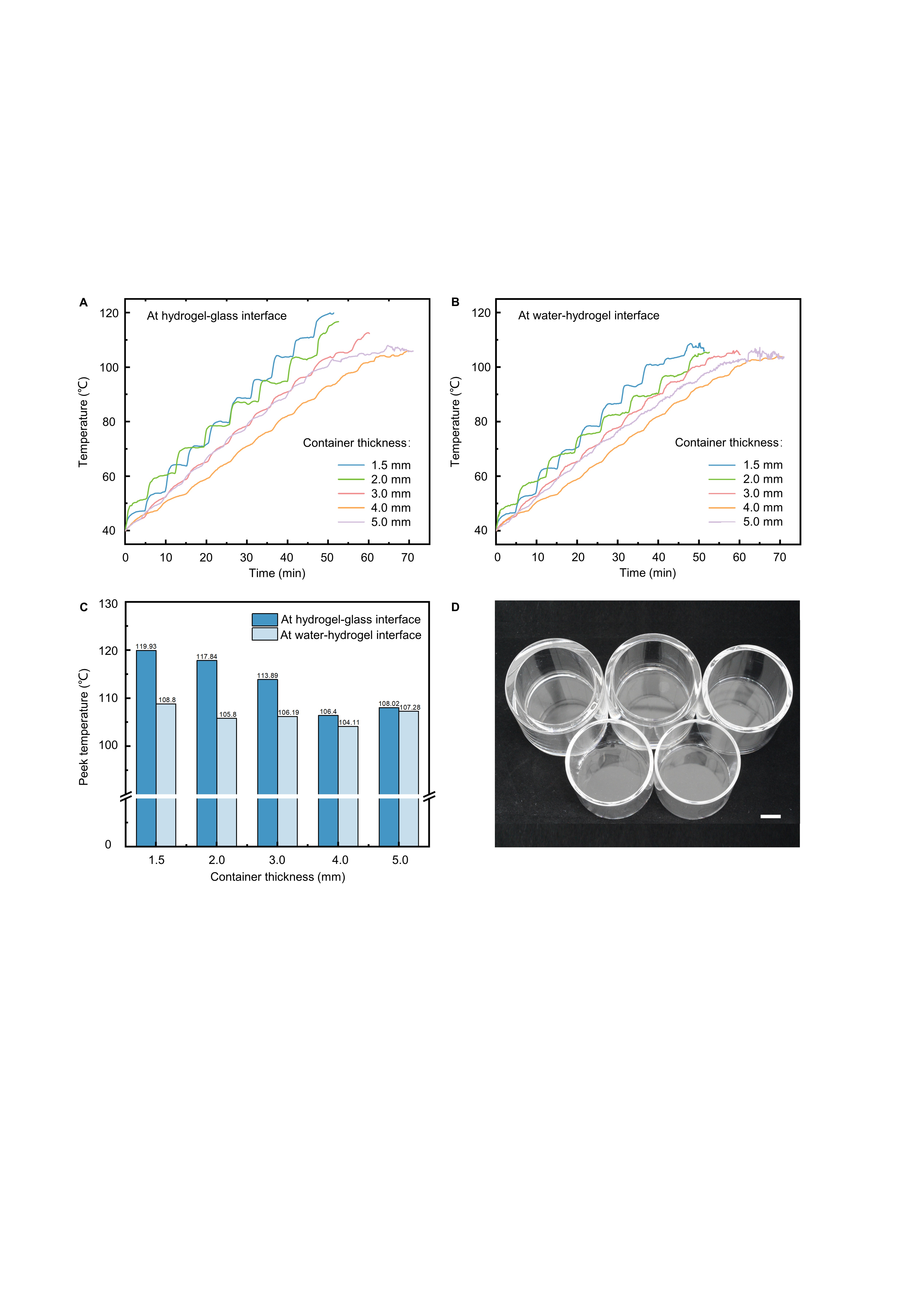


Fig. S11. The effect of container thickness on the hydrogel coating's ability to suppress boiling nucleation. (A-B) Temperature changes at the water-hydrogel interface and the hydrogel-glass interface during heating experiments. (C) The peak temperatures at the water-hydrogel interface and the hydrogel-glass interface during heating water in containers of 1.5mm, 2mm, 3mm, 4mm, and 5mm wall thickness. (D) Glass experimental containers with different wall thicknesses, inner diameter 40mm, depth 55mm. The Scale bar represents 1 cm.


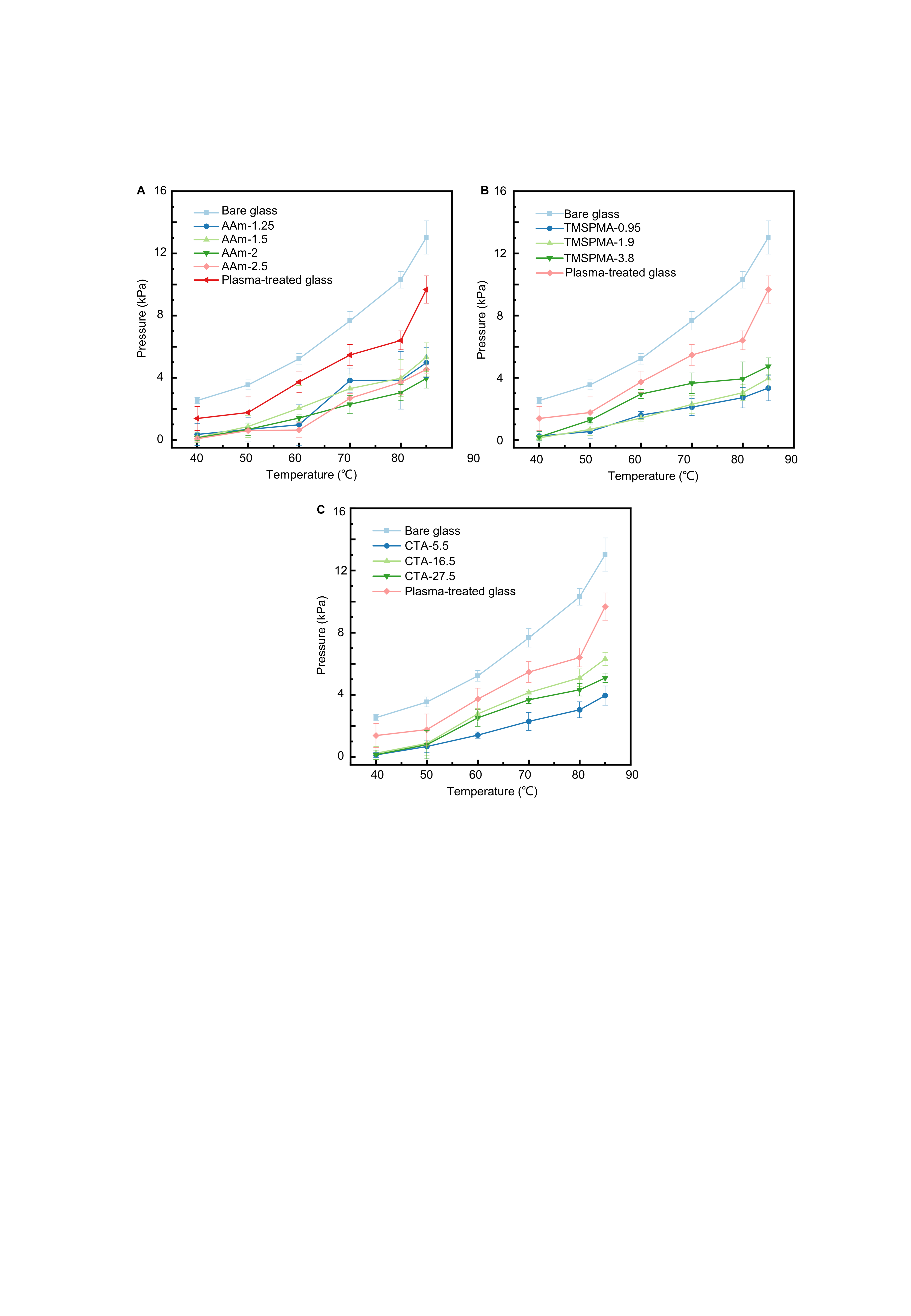


Fig. S12. Effects of hydrogel coating features on cavitation pressure. The effect of (A) water content, (B) polymer chain length, and (C) crosslink density of the hydrogel coating on cavitation pressure. Though the modulus and the toughness of hydrogel vary with those features, the surface properties of the hydrogel are not much changed, and the cavitation pressure shows no obvious relation to them.

References and Notes

1. T.D. Wheeler, A.D. Stroock, The transpiration of water at negative pressures in a synthetic tree, Nature, 455 (2008) 208-212.

2. H.J. Maris, Introduction to the physics of nucleation, Comptes Rendus Physique, 7 (2006) 946-958.

3. J. Tang, J. Li, J.J. Vlassak, Z. Suo, Fatigue fracture of hydrogels, Extreme Mechanics Letters, 10 (2017) 24-31.

4. J.A. Zimberlin, A.J. Crosby, Water cavitation of hydrogels, Journal of Polymer Science Part B: Polymer Physics, 48 (2010) 1423-1427.

5. J. Liu, S. Qu, Z. Suo, W. Yang, Functional hydrogel coatings, National Science Review, 8 (2021) nwaa254.

**Captions for supplementary movies**

**Movie S1.** Water cavitation on the surfaces of bare, plasma-treated, and hydrogel-coated glass by negative pressure

**Movie S2.** Cavitation occurs along the hydrogel-substrate interface when the hydrogel coating is not adhered to the substrate

**Movie S3.** Water boiling on a glass that is half-coated by hydrogel

**Movie S4.** Growth of boiling bubbles along the water-hydrogel, the hydrogel-glass and the water-glass interfaces during heating

**Movie S5.** Acceleration-induced cavitation and damage in bottles without hydrogel coating

**Movie S6.** Acceleration-induced cavitation and damage in bottles with hydrogel coating

**Movie S7.** Contact angle of water on hydrogel-coated glass
